# Risk factors for intensive care unit admission following correction surgery for adult spinal deformity

**DOI:** 10.1186/s13018-023-04227-0

**Published:** 2023-10-04

**Authors:** Chenkai Li, You Du, Shengru Wang, Jianguo Zhang, Yang Yang, Yiwei Zhao, Haoran Zhang, Xiaohan Ye

**Affiliations:** Department of Orthopedics, Peking Union Medical College Hospital, 1st Shuai Fu Yuan, Dongcheng District, Beijing, 100730 People’s Republic of China

**Keywords:** Risk factors, Deformity, Corrective surgery, ICU

## Abstract

**Objective:**

The literature currently available on the characteristics of patients who require intensive care unit (ICU) admission after correction surgery for adult spinal deformity is lacking; this study aimed to identify risk factors for postoperative ICU admission following correction surgery for adult spinal deformity.

**Methods:**

A retrospective review of patients who underwent primary posterior-based spinal fusion from 2015 to 2023 was performed. According to the ward they returned to, patients were further divided into an ICU group and a non-ICU group. Univariate and multivariate analyses were performed to evaluate preoperative and perioperative parameters to identify independent risk factors for postoperative ICU admission in adult spinal deformity patients.

**Results:**

A total of 274 patients were included, including 115 males (41.97%) and 159 females (58.03%). The mean age of the patients was 32.00 ± 11.16 years (19–77 years). Following adjusted analysis, the preoperative and perioperative factors that were independently associated with ICU admission were age, body mass index ≥ 28 kg/m^2^, neuromuscular spinal deformity, respiratory disease, grade III-IV American Society of Anesthesiologists (ASA) classification, a scoliosis Cobb angle ≥ 90°, a kyphosis Cobb angle ≥ 90°, and ≥ 12 fused segments. Compared with the non-ICU group, the ICU group had a higher incidence of complications, a longer hospital stay, and higher medical costs (*P* < 0.05).

**Conclusion:**

This study identified independent risk factors associated with postoperative ICU admission in adult spinal deformity patients; and explored relative measures to decrease or avoid the risk of postoperative ICU admission. Surgeons could use these data to develop and plan appropriate perioperative care processes in advance and provide consultation for family members before surgery.

## Background

Adult spinal deformity is defined as a heterogeneous spectrum of abnormalities of the thoracolumbar or lumbar spine occurring in adults [1, 2]. The etiologies of such deformities vary and consist of de novo onset, the progression of existing deformities or iatrogenic injury [1, 3]. Patients with severe spinal deformity may suffer from back pain, pulmonary dysfunction, and even neurological deficits. Conservative treatment for patients with a higher curvature degree is often ineffective, and most patients ultimately require surgical intervention. The primary goal of corrective surgery is to halt progression of the curve while simultaneously achieving permanent improvement of the deformity [4]. Compared with adolescent spinal deformities, adult spinal deformities are usually more severe and rigid. Therefore, to achieve ideal clinical outcomes, extensive soft-tissue releases, high-grade osteotomies, and long segmental fusions are often required during surgery, which lead to a prolonged operation time and a high volume of blood loss. Although patients can benefit tremendously from surgery, the risks of perioperative complications, a prolonged hospital stay, and extensive medical costs are also increased. In particular, to provide efficient recovery and decrease morbidity and mortality, some patients may be transferred to the intensive care unit (ICU) postoperatively. However, admission to the ICU is not necessary for all adult spinal deformity patients. It has been reported that ICU admission may be required in 15%-35% of pediatric patients [5, 6]. Currently, the literature available on the characteristics of patients who require ICU admission following corrective surgery for adult spinal deformity is lacking. To avoid the utilization of unnecessary health care resources, it is meaningful to identify the risk factors for ICU admission following the correction of adult spinal deformity. Therefore, in this study, we aimed to identify risk factors for ICU admission following the correction of adult spinal deformity.

## Materials and methods

A retrospective review of spinal deformity patients undergoing primary spinal fusion by instrumentation at the Peking Union Medical College Hospital between 2015 and 2023 was conducted. The study was approved by the hospital’s ethics committee. The inclusion criteria were as follows: ① patients aged > 18 years old; ② patients undergoing primary spinal fusion; and ③ patients for whom a posterior approach would be used. According to the ward they returned to, patients were further divided into an ICU group and a non-ICU group.

### Perioperative care

All of the patients received general anesthesia using the total intravenous anesthesia technique. The surgical procedure consisted of a posterior approach to the spine, pedicle screw implantation, osteotomy, and vertebral derotation. Neuromonitoring with motor and somatosensory-evoked potentials was routinely applied, combined with Stagnara’s wake-up test if necessary. Standard practice was to attempt extubation of all patients after surgery and patients would return to the general ward as long as their general condition was stable. Patients with an unstable general condition or delayed extubation were transferred to the ICU for continuous general treatment. When patients had stable hemodynamics and respiratory conditions without any medical support, they returned to the general ward.

### Preoperative parameters

Preoperative parameters included demographic data, spinal curve type, deformity etiology, smoking status, drinking status, the presence of comorbidities, ASA classification, preoperative hemoglobin (Hb) concentration (g/L), and Cobb angle (°). Demographic data included sex, age (years) and body mass index (BMI) (kg/m^2^). The type of spinal curve consisted of scoliosis, kyphosis, or kyphoscoliosis. The deformity etiology included idiopathic, congenital, neuromuscular, syndromic, and other etiologies.

### Perioperative parameters

Perioperative parameters included surgery-related parameters, major complications, length of hospital stay, ICU-related parameters, and medical costs. Surgery-related parameters consisted of the operation time (min), the blood loss volume (ml), the usage of red blood cells (U) or plasma (ml), fusion extension, pelvic fixation, and osteotomy type. Parameters related to hospital stay consisted of the number of hospitalization days and the number of postoperative hospitalization days. ICU-related parameters included the length of ICU stay (hours) and the time of extubation (hours).

### Clarification

To simplify the data analysis, the grouping criteria listed below were used:

1. BMI was used to further divide patients into four groups: < 18.5 kg/m^2^, 18.5–23.9 kg/m^2^, 24–27.9 kg/m^2^ and ≥ 28 kg/m^2^;
2. The secondary spinal deformity mainly included ankylosing spondylitis, tuberculosis, Scheuermann’s disease, and iatrogenic deformity;
3. Respiratory dysfunction was defined as a forced vital capacity (FVC) < 79% [7];
4. ASA classification was used to further divide patients into two subgroups: ASA classification I-II and ASA classification III-IV [6];
5. Cobb angle severity: a Cobb angle ≥ 90° was defined as severe spinal deformity [7];
6. Length of surgery: a prolonged operation was defined as an operation time ≥ 270 min [8];
7. Fusion extension: long fusion was defined as ≥ 12 fused segments [9];
8. Osteotomy type: grade I-II grade osteotomy was defined as low-grade osteotomy and > grade II osteotomy was defined as high-grade osteotomy [10];
9. Major complications were based on previously published studies, including neurological, respiratory, hematological, gastrointestinal, and hemodynamic complications, as well as infection [7];
10. Time of extubation: late extubation was defined as extubation > 24 h after the operation [7].

### Statistical analysis

Statistical analysis was performed using the Statistical Package for the Social Sciences (SPSS) 19.0 (SPSS, Inc., Chicago, IL, USA). In preliminary univariate analyses, a chi-square test and Student’s t test were used for perioperative factors. The chi-squared test was used for categorical variables, and Student’s t test was used for continuous variables. All variables with a P value < 0.1 were added into a multivariate logistic regression model to identify independent predictors associated with ICU admission. For all statistical purposes, a P value of less than 0.05 was considered significant.

## Results

We collected data from 274 patients who underwent posterior correction surgery between 2015 and 2023. All of the patients met the study criteria. There were 221 patients in the non-ICU group and 53 patients in the ICU group.

### Pre- and perioperative parameters

The mean age of the patients was 32.00 ± 11.16 years (19–77 years) and the mean BMI was 22.82 ± 5.37 kg/m^2^(14.22–52.68 kg/m^2^). There were 115 males (41.97%) and 159 females (58.03%). Regarding the type of spinal curve, 129 patients (47.08%) had scoliosis, 74 patients (27.01%) had kyphosis, and 71 patients (25.91%) had kyphoscoliosis. Ninety-seven patients (35.40%) were diagnosed with adult idiopathic spinal deformities, 50 patients (18.25%) were diagnosed with congenital deformities, 19 patients (6.93%) were diagnosed with neuromuscular deformities, 12 patients (4.38%) were diagnosed with syndromic deformities, 12 patients (4.38%) were diagnosed with degenerative deformities, and 84 patients (30.66%) were diagnosed with secondary deformities, including 75 ankylosing spondylitis kyphosis, 6 with tuberculous kyphosis, 2 with Scheuermann’s disease, and 1 with iatrogenic kyphosis. Fifty-one patients (18.61%) had a history of smoking, and 34 patients (12.41%) had a history of drinking. Among the cohort, 21 patients (7.66%) had neuromuscular disease, 135 patients (49.27%) had respiratory disease, 17 patients (6.20%) had cardiovascular disease, 4 patients (1.46%) had endocrine disease, 2 patients (0.73%) had rheumatic immune disease, 1 patient (0.36%) had blood disease, and 4 patients (1.46%) had a history of tumors. There were 219 patients (79.93%) classified as ASA I-II, and 55 patients (20.07%) were classified as ASA III-IV. The mean preoperative hemoglobin concentration was 134.90 ± 13.30 g/L (98.00–168.00 g/L). The mean scoliosis and kyphosis Cobb angles were 66.28 ± 29.62° (20.00–154.60°) and 74.65 ± 25.13° (22.80–160.00°) respectively.

The mean operation time was 365.47 ± 111.00 min (150–745 min) and the mean blood loss volume was 795.84 ± 529.20 ml (150–2500 ml). The mean volume of red blood cells used was 1.65 ± 1.92 U (0–8 U), and the mean volume of plasma used was 222.12 ± 267.19 ml (0–1400 ml). The mean number of fused segments was 10.89 ± 3.22 (5–17), and 24 patients received pelvic fixation. There were 127 patients (46.35%) who underwent low-grade osteotomy and 147 patients (53.65%) who underwent high-grade osteotomy. Thirty-six patients experienced major complications, including 22 with transient neurological complications (8.03%), 13 with pleural effusions (4.74%), and 1 with deep infection (0.36%). The mean hospital stay was 16.00 ± 7.00 days (7–71 days), and the mean postoperative hospital stay was 11.00 ± 6.00 days (5–61 days). For patients transferred to the ICU, the mean ICU stay was 29.00 ± 24.00 h (13–144 h), with 48 with early extubation (90.57%) and 5 with late extubation (9.43%). The mean medical costs were 204,917.80 ± 61,031.13 (83,580.16–407,215.79) (Table 1).

**Table 1:** Demographics of patients

	Non-ICU	ICU	*P*
Major complication	18	18	< 0.0001
Neurological	13	9	
Pleural effusions	5	8	
Deep infection	0	1	
Medical costs	198,031.43 ± 54,089.58	233,502.72 ± 77,639.79	0.0001
Hospitalization days	14.00 ± 5.00	23.00 ± 11.00	< 0.0001
Postoperative hospitalization days	9.00 ± 4.00	15.00 ± 9.00	< 0.0001
Length of ICU stay (hours)	/	29.00 ± 24.00	
Time of extubation (hours)	/	13.00 ± 14.00	
Early extubation	/	48	
Late extubation	/	5	

Univariate analysis showed that there were significant differences regarding age, BMI, etiology, neuromuscular disease or respiratory disease comorbidities, ASA classification, scoliosis or kyphosis Cobb angle, blood loss volume, usage of red blood cells or plasma, number of fused segments, and pelvic fixation between the ICU group and the non-ICU group (*P*  < 0.1) (Tables 2). Compared with the non-ICU group, the ICU group had a higher incidence of complications, a longer hospital stay, and higher medical costs (*P* < 0.05) (Table 1).

**Table 2:** The results of univariate analysis

	Non-ICU	ICU	*P*
Gender			0.537
Male	95	20	
Female	126	33	
Age (yrs)	31.62 ± 10.14	33.49 ± 14.56	0.014
BMI (kg/m^2^)			0.002
< 18.5	47	20	
18.5–23.9	101	10	
24–27.9	41	15	
≥ 28	32	8	
Etiology			< 0.0001
Adult idiopathic	90	7	
Congenital	32	18	
Neuromuscular	7	12	
Syndromic	7	5	
Degenerative	9	3	
Secondary	76	8	
Smoking status	44	7	0.327
Drinking status	27	7	0.819
Comorbidity			
Neuromuscular	7	14	< 0.0001
Respiratory	96	39	< 0.0001
Cardiovascular	11	6	0.109
Endocrine	2	2	0.170
Rheumatic immune	1	1	0.350
Blood	1	0	> 0.999
Tumor	2	2	0.170
ASA classification			< 0.0001
I-II	207	12	
III-IV	14	41	
Preoperative Hb (g/L)	135.06 ± 13.17	134.00 ± 13.91	0.603
Scoliosis (°)			< 0.0001
< 90°	203	28	
≥ 90°	18	25	
Kyphosis (°)			< 0.0001
< 90°	199	27	
≥ 90°	22	26	
Operation time (min)			0.248
< 270 min	47	7	
≥ 270 min	174	46	
Blood loss volume (ml)	740.16 ± 497.33	1021.70 ± 590.93	0.0004
Blood transfusion			
Red blood cells (U)	1.37 ± 1.71	3.50 ± 1.90	< 0.0001
Plasma (ml)	176.47 ± 229.28	546.50 ± 256.57	< 0.0001
Fused segments			< 0.0001
< 12	202	8	
≥ 12	79	45	
Pelvic fixation	12	12	0.0004
Osteotomy type			> 0.999
I-II	123	29	
≥ III	98	24	

### Independent risk factors for ICU admission

Following adjustment in a multivariate logistic regression model, independent predictors associated with ICU admission following surgery were age (odds ratio [OR] 1.09 [95% CI 1.03–1.16]; *P*  = 0.006), BMI ≥ 28 kg/m^2^ [OR 9.45 [95% CI 1.88–37.24]; *P*  = 0.013), neuromuscular spinal deformity (OR 9.73 [95% CI 1.25–140.94]; *P*  = 0.032), respiratory disease (OR 6.05 [95% CI 1.10–33.25]; *P*  = 0.039), ASA classification III-IV (OR 27.36 [95% CI 7.37–101.54]; *P*  < 0.0001), a scoliosis Cobb angle ≥ 90° (OR 12.02 [95% CI 2.35–61.63]; *P*  = 0.003), a kyphosis Cobb angle ≥ 90° (OR 4.51 [95% CI 1.22–16.62]; *P*  = 0.024), and ≥ 12 fused segments (OR 7.47 [95% CI 1.17–47.70]; *P*  = 0.034) (Table 3).

**Table 3:** The results of multivariate analysis

Risk factor	Adjusted OR [95% CI]	*P*
Age	1.09 [1.03–1.16]	0.006
*BMI*		
18.5–23.9	Reference	-
< 18.5	1.34 [0.29–6.06]	0.708
24–27.9	1.49 [0.14–9.73]	0.739
≥ 28	9.45 [1.88–37.24]	0.013
*Etiology*		
Adult idiopathic	Reference	–
Congenital	1.72 [0.30–9.97]	0.540
Neuromuscular	9.73 [1.25–140.94]	0.032
Syndromic	1.21 [0.08–18.63]	0.892
Degenerative	0.08 [0.02–3.32]	0.184
Secondary	0.18 [0.02–1.87]	0.151
*Comorbidity*		
Neuromuscular	1.15 [0.12–11.14]	0.906
Respiratory	6.05 [1.10–33.25]	0.039
ASA classification	27.36 [7.37–101.54]	< 0.0001
I-II		
III-IV		
Scoliosis (°)	12.02 [2.35–61.63]	0.003
< 90°		
≥ 90°		
Kyphosis (°)	4.51 [1.22–16.62]	0.024
< 90°		
≥ 90°		
Blood loss volume (ml)	1.00 [0.998–1.001]	0.503
*Blood transfusion*		
Red blood cells (U)	1.04 [0.60–1.82]	0.706
Plasma (ml)	1.002 [0.998–1.006]	0.256
Fused segments	7.47 [1.17–47.70]	0.034
< 12		
≥ 12		
Pelvic fixation	7.22 [0.99–52.55]	0.051

## Discussion

The recent data available on the characteristics of adult spinal deformity patients who required ICU admission following scoliosis surgery are limited. Therefore, the present study analyzed the independent risk factors for ICU admission following correction surgery for adult spinal deformity. The results of the multivariate analysis showed that ICU admission was required in patients with older age, BMI ≥ 28 kg/m^2^, neuromuscular deformities, respiratory dysfunction, ASA classification III-IV, a larger preoperative Cobb angle (scoliosis or kyphosis Cobb angle ≥ 90°), and a higher number of fused segments (≥ 12). At the same time, in this population, the patients had a higher incidence rate of perioperative major complications, a longer hospital stay, and higher medical costs.

It was easy to understand that age, BMI and ASA classification were associated with postoperative ICU admission. In addition to the severity of deformities progressing with age, compared with younger patients, older patients were prone to have more comorbidities. Thus, older patients usually have a higher ASA classification, which indicates a high risk of surgery. Moreover, if patients had higher BMI, it would increase the difficulty of surgical exposure leading to more blood loss and longer surgical time, which also increased the risk of ICU admission. A previous study reported that postoperative ICU admission was highly associated with the extent of central neuromotor impairment in pediatric patients [11]. Similarly, our results showed that patients diagnosed with neuromuscular spinal deformities were at high-risk for ICU admission. Possible reasons were that neuromuscular spinal deformities were more likely to progress into severe spinal deformities due to neuromuscular defects. As age increased, spinal deformities progressed. Besides, for patients diagnosed with neuromuscular spinal deformities, when respiratory muscles were affected, it also had negative impact on respiratory function. Thus, the difficulty and risk of surgery also increased. At the same time, it has been reported that the preoperative Cobb angle and a higher number of fused segments (≥ 12) could be risk factors for ICU admission [10, 12]. Compared with adolescent patients, due to severe and rigid curves, adult patients may need a more aggressive treatment strategy with multiple spinal levels in one session, such as a higher grade of osteotomy and a longer extent of fusion, which means a prolonged surgical time and increased intraoperative blood loss. Therefore, the risk of postoperative ICU admission and the incidence of complications was increased. Shaw et al. [11] demonstrated that the risk of in-hospital complications was significantly higher in the pediatric ICU patients than in patients in the hospital ward. In our study, the incidence rate of perioperative complications in ICU patients was 34.0%, which was significantly higher than in patients in the general hospital ward (8.1%). The major complications in ICU patients consisted of 9 neurological complications (50%), 8 pleural effusions (44.4%), and 1 deep infection (5.6%).

In addition, our results found that preoperative respiratory dysfunction was an independent risk factor for ICU admission. Patients with severe restrictive pulmonary dysfunction had a higher risk of being transferred to the ICU postoperatively. Lao et al. [13] demonstrated that in patients with extremely severe spinal deformities, respiratory function was negatively affected. Spinal deformities had a negative influence on the development of the thoracic cavity and lungs, which eventually impacted respiratory function, leading to hypoxemia, pulmonary hypertension, cor pulmonale, and hypercapnia. However, there was no consensus about the relationship between preoperative respiratory function and ICU admission [14–16]. Yuan et al. [17] conducted a study including 125 patients and found that the most strongly predictive factors for prolonged ventilation were a diagnosis of neuromuscular disease, an FEV1 < 40% and a VC < 60%. Gurajala et al. [18] claimed that preoperative pulmonary function test results were not associated with postoperative ICU admission. The main reason why preoperative pulmonary function could not predict the risk of postoperative ICU admission was that some patients could not adequately cooperate with the maneuvers required for respiratory function tests. It had been reported that 10% of primary scoliosis patients and nearly two-thirds of secondary scoliosis patients cannot provide any qualified respiratory function test results [16]. Thus, most studies only consisted of patients who could adequately cooperate with the test. However, these patients tended to have more complicated medical histories and were more likely to require postoperative mechanical ventilation and ICU admission. Therefore, the influence of preoperative pulmonary function was sometimes underestimated.

Postoperative ICU admission prolonged the postoperative hospital stay, resulting in an increase in the overall number of hospitalization days and medical costs. Shan et al. [4] reported that compared with patients initial treatment in the ICU, treatment outside the ICU could significantly decrease hospital charges. The authors claimed that medical costs could be reduced by 16% for patients who were not transferred to the ICU. Kamerlink et al. [19] assessed the proportion of medical costs, including charges incurred during the surgical process, operating- room costs and hospital charges for the treatment of adolescent idiopathic scoliosis. The results showed that general hospital ward and ICU charges accounted for 22% of the total medical costs, only secondary to implant costs (29%). Therefore, patients should avoid going to the ICU postoperatively as much as possible. To avoid postoperative ICU admission, several management strategies could be applied. First, for patients with multiple comorbidities, preoperative multidisciplinary treatment is necessary; Second, for patients with poor conditions, perioperative nutritional support or respiratory exercise should be performed to improve their condition and decrease their surgical risk. Third, for patients with severe spinal deformities, traction before surgery or staged surgery could be the optimal treatment strategy [9]. Forth, a previous study had demonstrated that the anesthetic regimen was significantly correlated with late extubation [7]. Thus, it is essential to select rapid-acting anesthetics to decrease the effects of respiratory depression, which would ultimately reduce the anesthesia and surgical times. Finally, it is meaningful to equip skilled medical staff to solve any change in a patient’s condition [7].

This study also had several limitations. First, this was a retrospective study with a relatively small sample size. Second, intra- and postoperative care as well as anesthetic techniques have been developed over the years, which might have resulted in different outcomes. Third, this study only involved a single surgeon, and the technology, surgical skill, and experience of the surgeon evolved over a decade. Moreover, different surgeons also have different surgical skill levels. Third, we only evaluated data from a single center, and variations in the anesthetic regimen, surgical strategy, extubation criteria, and ICU admission criteria could also lead to different results. Therefore, our results may not be generalizable to other institutions. Finally, this study only considered the posterior approach, and the assessment of the relationship between surgical approaches and postoperative ICU admission was not sufficient.

## Conclusion

Patients who were transferred to the ICU postoperatively had a higher incidence rate of perioperative major complications, a longer hospital stay, and higher medical costs. Thus, it is important to identify the risk factors for postoperative ICU admission following corrective surgery for adult spinal deformity. Surgeons should be cautious when patients are older, BMI ≥ 28 kg/m^2^, have neuromuscular deformities, have respiratory dysfunction, are classified as ASA III-IV, have a larger preoperative Cobb angle (scoliosis or kyphosis ≥ 90°), or have a higher number of fused segments (≥ 12). At the same time, comprehensive preoperative evaluation and preparation, cautious surgical performance, and appropriate perioperative management are necessary to decrease the risk of postoperative ICU admission.

## Data Availability

The final dataset will be available from the corresponding author.

## Abbreviations

ICU: Intensive care unit
ASA: American society of anesthesiologists
Hb: Hemoglobin
BMI: Body mass index
FVC: Forced vital capacity
